# Traumatism as an Antiological Factor in Appendicitis

**Published:** 1907-09

**Authors:** 


### TRAUMATISM AS AN ANTIOLOGICAL FACTOR IN APPENDICITIS.

---

Jno. B. Deaver has made an extensive review of the literature and from it deduces the following conclusions:

I would like to call attention to the following facts, which seem to be of most importance in considering the relationship of trauma and acute appendicitis:

(1). From personal experience and from a study of the cases found in the literature I do not consider that trauma is ever the direct exciting cause of acute appendicitis in a perfectly normal appendix.

(2). I believe that an acute attack of appendicitis can follow a severe blow upon the abdomen or fall upon the abdomen, or due to muscular contractions of the ileopsoas muscle in an appendix which has been previously inflamed only under the following conditions:

A. In a latent or residual abscess or extensive pathological lesion of the appendix, where the appendix did not occupy a deep pelvic position, but is in close proximity to the anterior abdominal wall, esvere direct traumatism may precipitate an acute attack.

B. Strong contractions of the ileopsoas muscle cannot in my opinion be the immediate cause of an acute attack of appendicitis, where the appendix is chronically diseased where it has extensive pathological lesions, unless it is firmly adherent to and not simply in apposition to the peritoneum overlying this muscle.

C. The degree of traumatism to be a factor in the causation of appendicitis must be direct and of considerable force; such force applied to the right iliac fossa may tear the underlying parietal peritonaeum and so simulate an acute attack of appendicitis that only opening the abdomen and exposing the appendix could definitely settle the matter.

(3). The acute attack of appendicitis of traumatic origin is observed more frequently in males than in females on account of their more active life and greater liability to injury and strains, and between the ages of ten and twenty-five years.

(4). In an appendix previously diseased the liability to an acute attack of appendicitis supervening upon injury is in direct ratio to the degree of injury, and depends entirely upon the pathological changes present in the appendix at the time of injury.

(5) I maintain that it is exceedingly rare to find a case of acute appendicitis in which it can be definitely stated that traumatism is the direct exciting factor. This statement is borne out by a review of 1,400 cases seen at the German Hospital during the years 1904, 1905, and 1906, and of this number in only one patient was there any history of injury at all, and in this case it was questionable whether the injury had anything to do in causing the attack. The history of the case is as follows:

J. H., age twenty-four years, admitted to the German Hospital, July 7th, 1905. Three months before admission, while plowing, the plow brake pulled forward, striking the patient's abdomen with the plow handle. The patient did not at that time have any pain in the abdomen, but several hours later pain developed in the right side over the appendix, with some nausea, and it became impossible for the patient to lift anything. This condition lasted one day, and then disappeared. On June 30th, while boarding a train he felt a stitch in the right side of the abdomen over appendix like something giving away. This pain was acute, without nausea or vomiting. The pain was easier on flexing his legs on abdomen. Examination showed rigidity of right rectus; acute tenderness over McBurney's point. At operation the appendix was found acutely inflamed.

In a review of the American, English, French, German, and Italian literature, it is a remarkable fact that of the great number of cases of acute appendicitis reported during the past forty to fifty years only one hundred and thirty cases can be found in which traumatism has any bearing whatever, and of these one hundred and thirty cases only thirteen cases can be directly attributable to traumatism for the production of the acute attack. In seventy-seven cases the previous history is not recorded, and in thirty-five cases the previous history is negative.

(6). The mortality is very high in these cases on account of (a) the failure to recognize the conditions until the disease is well advanced; (b) the rapid gangrene and perforation which occurs; and (c) the delay in operation.

(7). I must strongly urge a more careful study of this class of cases and, as soon as a diagnosis has been made, insist upon an immediate operation if we are to obtain the best results.—New York Medical Journal, June 15th, 1907.

---

The neutrality and general purity of the salts entering the composition of Peacock's Bromides have been attested to by eminent chemists. This assurance in its purity and uniformity is of great moment to the general practitioner when he desire to employ a continuous bromide treatment. It is a palatable preparation and as each fluid ounce contains fifteen grains of the combined bromides the dose is easily adjusted.

